# Discharge of late preterm newborn: appropriated, controlled…namely safe

**DOI:** 10.1186/1824-7288-40-S2-A7

**Published:** 2014-10-09

**Authors:** A Coscia, A Soldi, C Perathoner, L Occhi, E Bertino

**Affiliations:** 1 SC Neonatology, University of Turin, AOA Città della Salute e della Scienza, Turin, Italy

## 

Late-preterm newborns accounted for 8.7% of all US births in 2009, while in Italy, according to Euro-Peristat Report 2010, rate of preterm live births between 32 and 36 weeks accounts for 6.4%: therefore late-preterm incidence is around 5% [1]. In the literature is reported that late preterm infants are at increased risk of neonatal mortality and morbidity, including feeding problems, hyperbilirubinemia, hypoglycemia, and respiratory problems. So, in recent years, research has focused on hospital care, with little known about the real needs of care after discharge and in the home setting. However, it’s known that early discharge places these infants at greater risk of complications such as rehospitalizazion, particularly in breastfed infants [2].

Therefore in this population it’s fundamental to plan an “appropriate” discharge. What does “appropriateness” mean? In health care the appropriateness has two aspects: 1) the “clinical” appropriateness that refers to the criteria of efficacy and safety ; 2) the so-called “administrative” appropriateness that indicates the extent of provision of health according to the criterion of efficiency, that is the best use of available resources, with respect to the clinical case to be treated. Because the resources available vary by context, administrative appropriateness is a very dynamic concept.

In the discharge of late-preterm baby, clinical appropriateness requires individualization and involvement of family. Discharge criteria are substantially similar to those of full-term [3] but include longer observation times, more attention to the real understanding and involvement of the family in the scheme of nutrition and follow-up, and an increased need for planning follow-up and integration with local services.

Although discharge criteria for late preterm infants are quite precise, however there is a large inter-center heterogeneity regarding the timing of discharge. It’s clear that the choices on the discharge of late preterm newborns are strongly influenced by the organizational context. It should be essential to have accurate population-based surveillance data and organizational data, as well as clinical ones. Only in this way it is possible to evaluate the efficacy (and on which outcomes) of programs of protected discharge, and their compatibility with the available resources. For example, some studies suggest that home visiting promotes improved parent-infant interaction; however further studies are needed to demonstrate whether such interventions in at-risk populations may strengthen their impact and cost benefits [4].
